# Advancements in cardiac structures segmentation: a comprehensive systematic review of deep learning in CT imaging

**DOI:** 10.3389/fcvm.2024.1323461

**Published:** 2024-01-22

**Authors:** Turki Nasser Alnasser, Lojain Abdulaal, Ahmed Maiter, Michael Sharkey, Krit Dwivedi, Mahan Salehi, Pankaj Garg, Andrew James Swift, Samer Alabed

**Affiliations:** ^1^ Department of Infection, Immunity and Cardiovascular Disease, The University of Sheffield, Sheffield, United Kingdom; ^2^ College of Applied Medical Science, King Saud bin Abdulaziz University for Health Science, Riyadh, Saudi Arabia; ^3^ Department of Clinical Radiology, Sheffield Teaching Hospitals, Sheffield, United Kingdom; ^4^ Norwich Medical School, Faculty of Medicine and Health Sciences, University of East Anglia, Norwich, United Kingdom; ^5^ Insigneo Institute, Faculty of Engineering, The University of Sheffield, Sheffield, United Kingdom

**Keywords:** artificial intelligence, machine learning, deep learning, cardiac CT, segmentation, quality, systematic review

## Abstract

**Background:**

Segmentation of cardiac structures is an important step in evaluation of the heart on imaging. There has been growing interest in how artificial intelligence (AI) methods—particularly deep learning (DL)—can be used to automate this process. Existing AI approaches to cardiac segmentation have mostly focused on cardiac MRI. This systematic review aimed to appraise the performance and quality of supervised DL tools for the segmentation of cardiac structures on CT.

**Methods:**

Embase and Medline databases were searched to identify related studies from January 1, 2013 to December 4, 2023. Original research studies published in peer-reviewed journals after January 1, 2013 were eligible for inclusion if they presented supervised DL-based tools for the segmentation of cardiac structures and non-coronary great vessels on CT. The data extracted from eligible studies included information about cardiac structure(s) being segmented, study location, DL architectures and reported performance metrics such as the Dice similarity coefficient (DSC). The quality of the included studies was assessed using the Checklist for Artificial Intelligence in Medical Imaging (CLAIM).

**Results:**

18 studies published after 2020 were included. The DSC scores median achieved for the most commonly segmented structures were left atrium (0.88, IQR 0.83–0.91), left ventricle (0.91, IQR 0.89–0.94), left ventricle myocardium (0.83, IQR 0.82–0.92), right atrium (0.88, IQR 0.83–0.90), right ventricle (0.91, IQR 0.85–0.92), and pulmonary artery (0.92, IQR 0.87–0.93). Compliance of studies with CLAIM was variable. In particular, only 58% of studies showed compliance with dataset description criteria and most of the studies did not test or validate their models on external data (81%).

**Conclusion:**

Supervised DL has been applied to the segmentation of various cardiac structures on CT. Most showed similar performance as measured by DSC values. Existing studies have been limited by the size and nature of the training datasets, inconsistent descriptions of ground truth annotations and lack of testing in external data or clinical settings.

**Systematic Review Registration:**

[www.crd.york.ac.uk/prospero/], PROSPERO [CRD42023431113].

## Introduction

Cardiac imaging is playing an increasing role in the investigation and monitoring of cardiovascular disease (CVD), which remains the leading cause of mortality and morbidity worldwide with more than 17 million deaths per year. Computed tomography (CT) is the mainstay cross-sectional imaging modality of radiology departments and can provide useful information about the heart. Dedicated cardiac CT is performed for a range of indications, including assessment of coronary artery disease, valvulopathy, and congenital heart disease, as well as preoperative planning and postoperative follow-up. The heart can also be assessed on CT undertaken for non-cardiac indications—such as CT pulmonary angiography for pulmonary embolism or CT angiography for acute aortic syndrome—allowing the identification of incidental but potentially significant findings and cardiac complications of non-cardiac disease (1–5).

**Figure 1:**
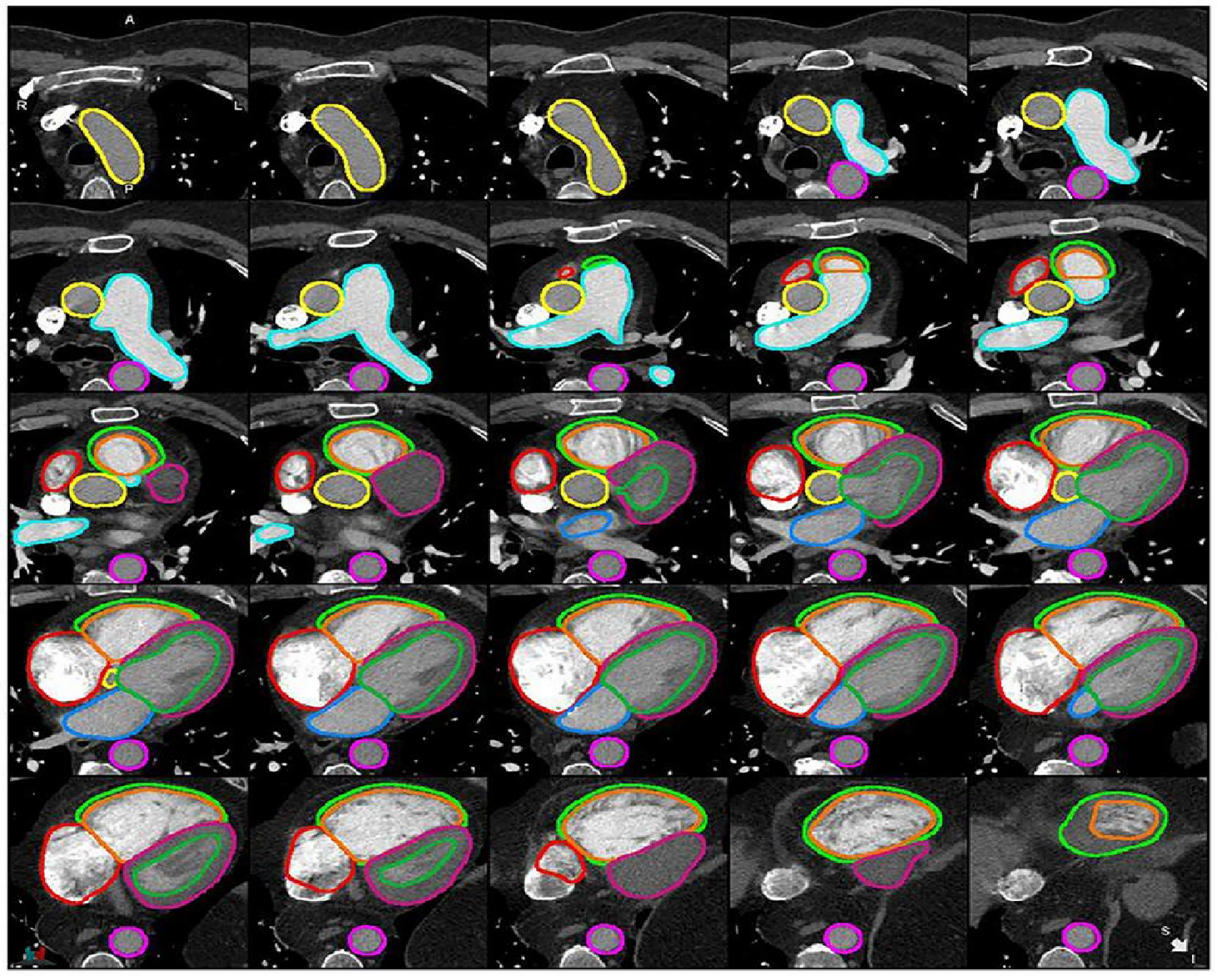
Example of cardiac structures’ segmentation on CTPA images (6). Ascending aorta (yellow), pulmonary artery (light blue), descending aorta (pink), right atrium (red), RV epicardium (light green), RV endocardium (orange), LV epicardium (purple), LV endocardium (green), left atrium (blue).

Segmentation refers to the process of delineating (or “contouring”) the edges of features on images. The features in question can be anatomical structures (such as the cardiac chambers) or pathological lesions (such as an area of myocardial infarction). Cardiac structures segmentation helps in the assessment of cardiac structures (Figure 1) and can assist with earlier detection of cardiac abnormalities (1, 5, 7–9). Traditionally, segmentation has been performed manually by radiologists. This process is time-consuming, subjective and prone to high levels of intra- and inter-observer variability. The application of artificial Intelligence (AI) to cardiac segmentation is of growing interest and offers potential improvements to efficiency and reliability compared to manual segmentation alone (10, 11). Furthermore, it was shown that the variability between observers and between an observer and an AI segmentation is similar (6). Recent years have seen a shift towards machine learning (ML) as the approach of choice for segmentation tasks. ML describes the process by which algorithms automatically identify patterns in data in order to make decisions or predictions when faced with new data. Deep learning (DL) is a subtype of ML in which multiple layers of algorithms—typically neural networks—are utilised, enabling the identification of more complex patterns and the generation of more accurate decisions (12, 13). Supervised DL depends on providing algorithms with accurate data for training, such as CT images labelled with manual segmentations by radiologists (6). Segmentation quality can be evaluated by comparing the degree of similarity between the DL and manual contours using either region-based indicators [such as the Dice similarity coefficient (DSC) or Jaccard index] or surface distance indicators (such as the Hausdorrf distance) (14).

This systematic review aimed to identify and appraise existing DL approaches to segmentation of cardiac structures on CT.

## Methods

The systematic review protocol was registered with The International Prospective Register of Systematic Reviews (PROSPERO; CRD42023431113). The systematic review was undertaken and is reported in line with the Preferred Reporting Items for Systematic Reviews and Meta-Analyses (PRISMA) guidance (15).

### Search strategy and inclusion criteria

Embase and Medline databases were searched from January 1, 2013 to December 4, 2023. The search strategy was developed around the key themes of the review: “AI”, “CT” and “cardiac”. The full search strategy is provided in Supplementary Table S1. Original research studies published after 1 January 2013 were eligible for inclusion if they (1) presented supervised DL-based tools (2) for segmentation of cardiac structures and non-coronary great vessels (3) on CT. Studies were excluded if they used non-English language, non-deep learning algorithms or other algorithms [e.g., graph-cut algorithm, principal component analysis (PCA), and Active shape model (ASM)].

### Screening and data extraction

Database results screening and full text assessment was undertaken by TA and confirmed by SA and AJS. Data extraction was performed by two investigators independently (TA and LA) using the same approach. The data extracted from each study included information about the study design and purpose (such as the type of cardiac structure being segmented), DL approach (such as method and architecture) and segmentation performance (such as reported DSC values). The classification models, which were employed to assign labels to CT images or specific areas of interest within those images to determine whether particular anatomical structures are present, and the segmentation models, which focus on precisely delineating the boundaries of these structures by labelling individual pixels that allows for the precise partitioning of CT images into distinct regions to represent anatomical structures, were extracted for each study (3, 8, 10). The country of each study was classified based on the location of the first author's primary institution. The performance of each DL tool was summarised based on the reported DSC; in cases where the DSC was not reported, it was calculated based on previously published formulas (4).

### Quality assessment

Each included study was assessed for compliance with the criteria of the Checklist for Artificial Intelligence in Medical Imaging (CLAIM) (16). The 42 individual criteria were divided into four domains: study description, dataset description, model description and model performance (17, 18).

## Results

### Study characteristics

**Figure 2:**
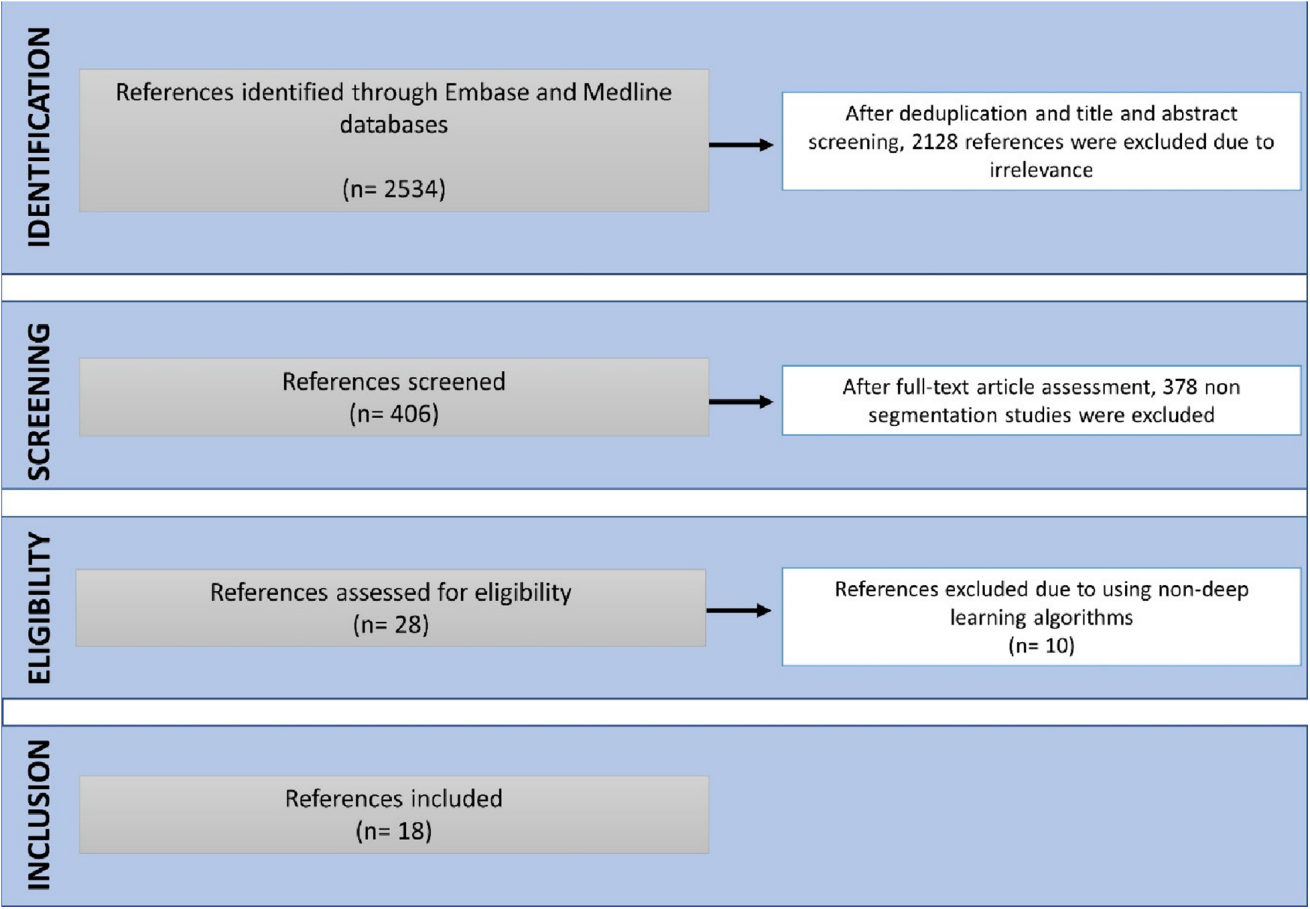
PRISMA graph shows the included studies.

**Figure 3:**
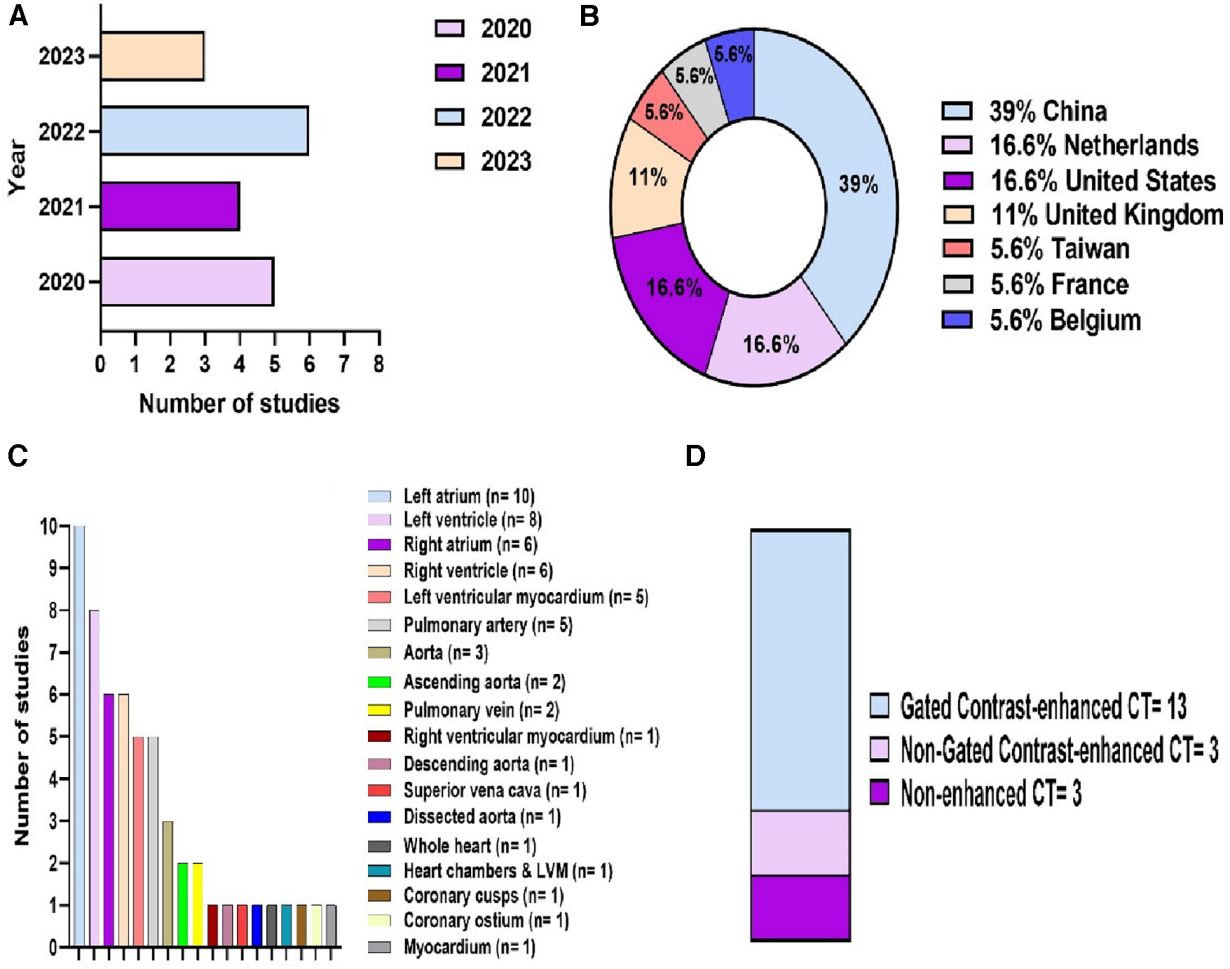
Descriptive information of the included studies. (**A**) Publications by year. (**B**) Location of the study. (**C**) Cardiac structures segmented. (**D**) CT acquisition used.

18 studies were eligible for inclusion (Figure 2). These were published between 2020 and 2023, with China having been the most common study location (39%) (Figures 3A,B). Half of the studies (9/18) segmented multiple structures (Table 1). The most frequently segmented cardiac structures were the left atrium (10/18) and left ventricle (8/18) (Figure 3C). Most studies (68%) used ECG-gated contrast-enhanced CT images to develop their models (Figure 3D).

**Table 1:** A summary of the included studies shows segmented cardiac structures, CT acquisition, models’ description, and used datasets for each study.

Study	Segmented structures	CT acquisition	Description			Dataset
			Classification model	Segmentation model	Dimension	
Abdulkareem et al. (3)	LA	CCT	ResNet50	U-Net	2D axial	Trained, validated, and tested with 150 patients
Aquino et al. (19)	LA, LV, LVM, RA, and RV	CTA	Not reported	I2I combined with cVAE	3D	Trained with 443 images, validated with 57 and tested with 55
Astdillo et al. (2)	Coronary cusps and Left and right coronary ostium	CCT	Not reported	CNNs	3D	Trained with 344 and validated with 100 patients
Bruns et al. (20)	LA, LV, LVM, RA, RV, Ascending aorta, and PA.	CT—& CCT	Not reported	CNNs	3D	Sixfold cross-validation (15 images for training and 3 for validation and testing)
Bruns et al. (7)	Heart chambers and LVM	CCT	Not reported	CNNs	3D	Development set = 14 patients Test set = 1,497 patients
Chen et al. (10)	LA	PVCT	ResNet50	U-Net	2D axial	Trained with 62, validated with 15, and tested with 20 images
Chen et al. (14)	Aorta	CCT & CTA	Not reported	nnU-Net	3D	Trained with 704 images and tested with 410 images
Guo et al. (1)	LVM	CCTA	Not reported	Deep attention U-Net combined with AGs	3D	Fivefold cross validation of 100 images
Guo et al. (21)	LV	CCT	Not reported	U-Net based on SS-BL-Net	3D	18 patients: each has 10 scans used for either training or testing with sixfold cross-validation
Gupta et al. (22)	LV	CTA	Not reported	Octree representation and octree-based CNN	3D	Fivefold cross-validation (160 images for training and 40 for validation)
Kazi et al. (23)	LA	CTA	Not reported	U-Net: unified-image-volume and regional patch-volumes	3D	Trained with 28 images and validated with 12 images
Li et al. (24)	LA and PV	LDCT	Not reported	CNNs—modified V-Net: GAB and SCAB	3D	68 CT images with fivefold cross-validation
Lyu et al. (4)	Dissected aorta	CTA	CNNs based on ResNet	CNNs based on PSPnet	2D and 3D	42 volumes sixfold cross-validation (5 groups for training and 1 for testing)
Oever et al. (11)	Whole heart, LA, LV, RA, and RV	LDCT	InceptionResNetV2	U-Net	2D three planes	Trained with 41, tuned with 3, and validated with 6 volumes
Sharkey et al. (6)	LA, LV, LVM, RA, RV, RVM, ascending aorta, descending aorta, and PA	CTPA	Not reported	nn-UNet	3D	Trained with 80, validated with 20, and tested with 100 internal and 20 external patients
Sharobeem et al. (8)	Aorta, coronary sinus, LA, LV, LVM, RA, RV, PA, PV, and SVC	CCT	CNNs based on SqueezeNet	CNNs based on DenseVnet	3D	Trained with 55, validated with 8 and tested with 8 patients
Yao et al. (25)	LA, LV, RA, RV, aorta, PA, and myocardium	CTA	Not reported	U-Net	2D and 3D	Fourfold cross-validation (51 images for training and 17 for testing)
Yuan et al. (5)	PA	CTPA	Not reported	PA-Net	2D axial	Trained with 30, validated with 10, and tested with 10 patients

CCT, cardiac CT; CTA, CT angiography; CCTA, coronary CT angiography; PVCT, pulmonary vein CT; LDCT, low dose CT; PSPnet, pyramid scene parsing network; AGs, attention gates; I2I, image to image; cVAE, conditional variational autoencoder; SS-BL-Net, spatial-sequential bi-directional learning network; GAB, grouped attention block; SCAB, spatial and channel attention block.

### DL architectures and models

The included studies used different DL architectures and models to segment the anatomical structures. Most of the studies used the Convolutional neural network (CNN) or U-shaped Neural Network (U-Net). Based on the classification and segmentation models’ definitions mentioned previously, a majority of the studies preferred to manually determine whether particular anatomical structures are present in the images or not (13/18) and the others used classification models to do instead (5/18). The algorithms of the included studies are summarised in Table 1.

### Segmentation performance

**Figure 4:**
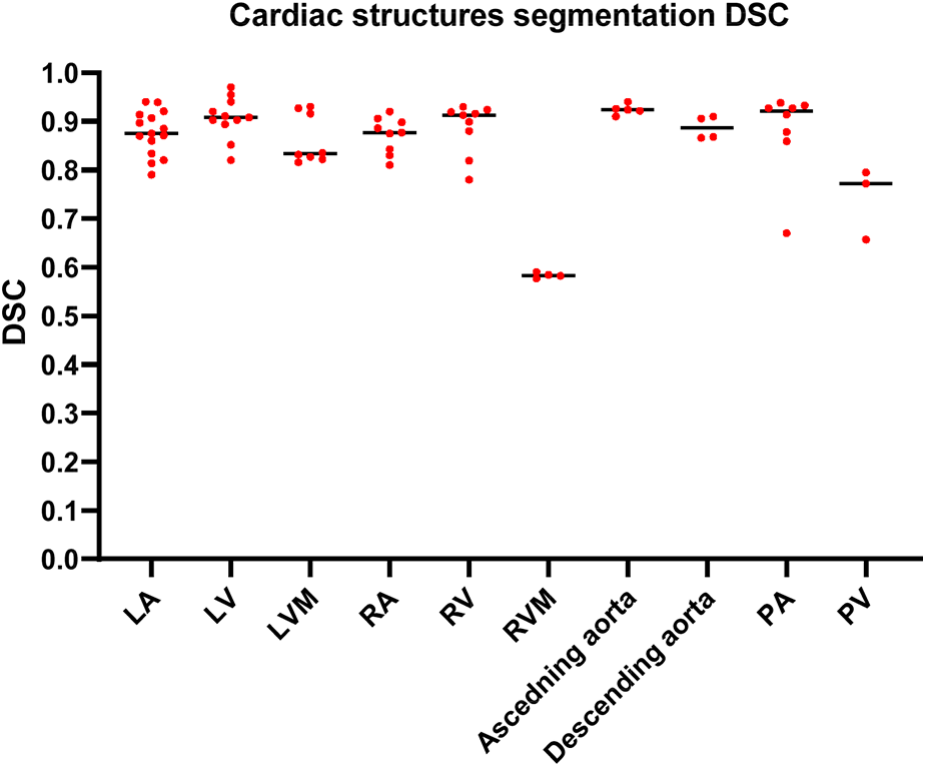
Column scatter plot showing DSC scores of different cardiac structures segmentation studies. Median DSC (solid line) is indicated.

The highest median DSC scores were achieved by PA (0.92, IQR 0.87–0.93) and ascending aorta segmentation studies (0.92, IQR 0.91–0.93), followed by LV and RV segmentation studies, (0.91, IQR 0.89–0.94) and (0.91, IQR 0.85–0.92), respectively. The lowest median DSC scores were found in RVM (0.58, IQR 0.57–0.59). A summary of the DSC scores for the most common segmented cardiac structure is shown in Figure 4. DSC scores of each cardiac structure shown in Supplementary Table S3 and Supplementary Figure S1.

### Quality reporting

**Figure 5:**
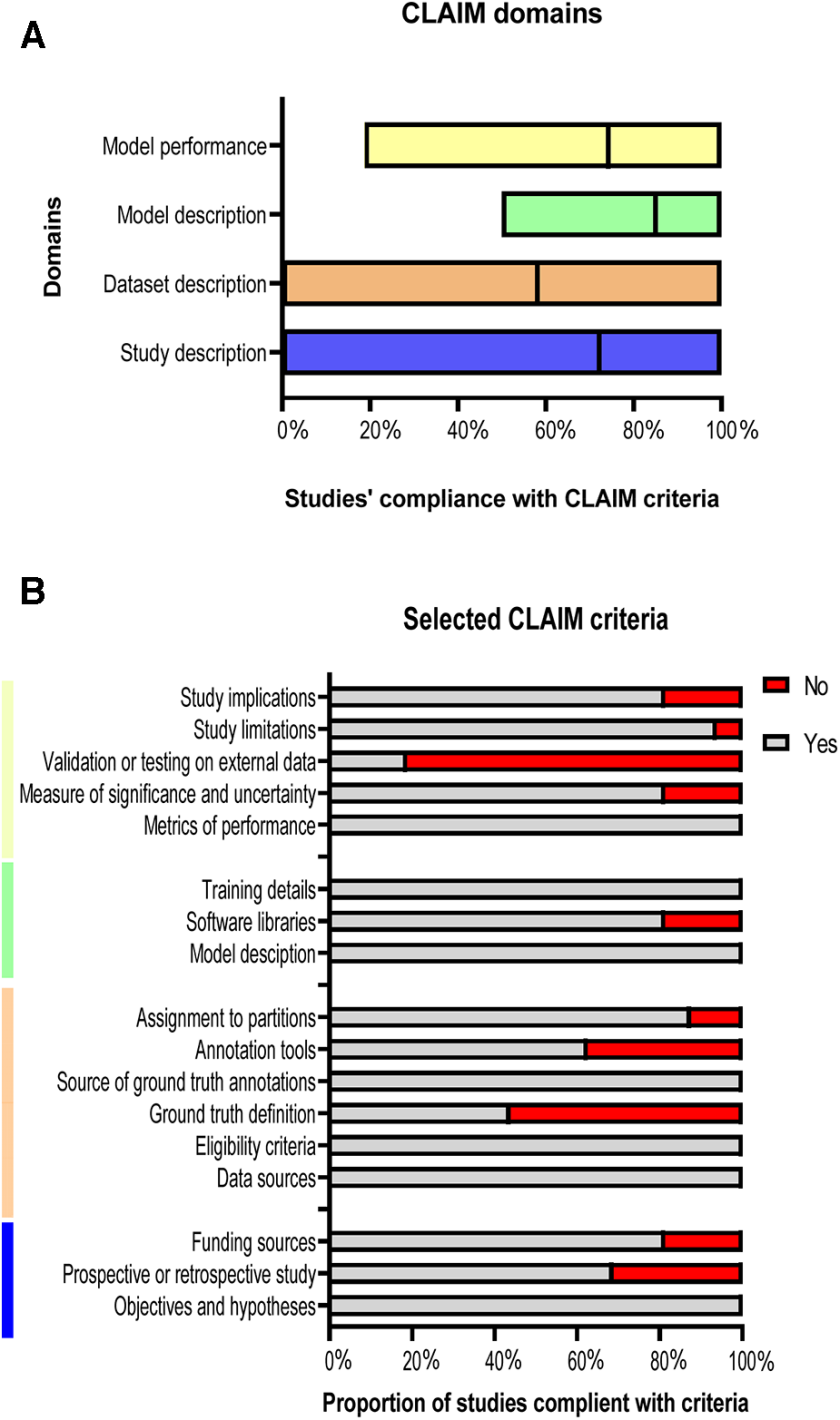
Studies’ compliance with CLAIM. (**A**) Floating bars present the compliance of the 18 included studies with the CLAIM, classified into four domains. The mean (solid line) is indicated. (**B**) Compliance of the studies with selected CLAIM criteria grouped by domains.

Compliance with the criteria of CLAIM is summarised in Figure 5. The included studies achieved a mean of 72% in study description criteria, 58% in dataset description, 85% in model description, and 74% in model performance (Figures 5A,B). A minority of studies tested or validated their models on external data (19%) or provided a clear description of the ground truth (44%). Furthermore, 37.5% of the studies have not stated the used annotation tools and the study design was clearly reported in only 69% of the studies. The quality assessments’ results for the whole studies are shown in Supplementary Table S2.

## Discussion

Segmentation is an important process in the evaluation of the heart on imaging. However, manual segmentation is time-consuming and may suffer from poor reproducibility, even when performed by experienced cardiac radiologists (1, 2, 14). In recent years, different DL applications have been developed to automate the segmentation of cardiac structures on imaging and may help to improve efficiency and reliability (1). In a previous systematic review in 2022, 209 studies were included for AI-based cardiac MRI segmentation (17, 18). However, our systematic review of DL-based cardiac CT segmentations identified only 18 studies. Due to the availability and speed of CT compared to other modalities, and its increased mention in cardiac disease management guidelines, automatic and accurate CT segmentation models for cardiac structures are likely to increasingly play a role in routine clinical use (2, 19). This systematic review identified 18 original research studies published since 2013 that presented DL tools for automated segmentation of cardiac structures on CT. The design, datasets, CT acquisitions, publication years, locations, algorithms and DSC scores of each study was appraised and the quality of reporting assessed according to compliance against CLAIM. DL showed high quality in segmenting various anatomical structures across different CT acquisitions. Even in non-gated contrast-enhanced CT acquisitions, such as CTPA, it continues to yield high DSC scores. Moreover, the included studies employed a variety of DL architectures and models, consistently achieving high DSC scores regardless of the specific network types employed. Nevertheless, a high DSC score is not the sole metric for assessing segmentation quality; other surface distance indicators, such as Hausdorff distance, should be measured to provide a more comprehensive assessment.

Despite the high repeatability of DL segmentation in conditions similar to the training data, its accuracy in different populations and image quality can vary. Two different studies using a similar framework of a ResNet50 classification model and an U-Net segmentation model showed different DSC scores (3, 10). The DSC score reached 0.96 when applied on PVCT images with a study population mean age of 54 ± 11 years (10) while achieving 0.89 on CCT images in an older population of 63 ± 10 years (3). Both datasets were atrial fibrillation patients, which may have affected imaging quality and despite this DSC scores remained high. Segmenting the LA in the absence of contrast was assessed in different segmentation and classification models however DSC score of 0.83 was the highest achieved and segmentation of low-dose and non-contrast acquisitions remains a challenge (11, 24). Another challenge is segmenting 3D volumetric images due to their size requiring large memory capacity. An octree-based representation for 3D CT images has been developed to overcome the limitations of sacrificing the information and resolution of 3D volumes. The developed model achieved a DSC score of 0.97 in LV segmentation by using a user-defined intensity tolerance to compress the 3D volumes before the segmentation step. As the tolerance increases, the compression will increase, but the DSC score will be negatively affected (22). In addition, the utilisation of a spatial sequential network (SS-Net) for unsupervised learning of the deformation and movement characteristics exhibited by the LV combined with sequential contextual data obtained from bidirectional learning showed a DSC score of 0.96 in LV segmentation (21). In this method, the image sequence is explored in two directions (i.e., chronological and reverse-chronological).

Defining the inner and outer borders of the LV myocardium has been achieved using five different segmentation models. A combination of image-to-image (I2I) segmentation network with conditional variational autoencoder (cVAE) had the highest DSC score (19), followed by a 3D segmentation U-Net model using deep attention network (Attention Gates) to selectively emphasise the myocardium boundary structures (1). In contrast, only one study focused on segmenting the more complex and thinner RV myocardium. Sharkey 2022 (6) developed two nn-UNet models on CTPA images by first implementing a low-resolution model to localise the cardiac structures followed by a high-resolution model extracting different features from the localised image. The DSC score achieved remained low (i.e., 0.59), however there was substantial discrepancy in RV myocardium measurements between the manual observers themselves. Therefore, interpreting a low DSC score is challenging when the ground truth contains inherent noise. In addition, the models were applied on non-gated CTPA images, and therefore not comparable to the quality of gated acquisitions (6).

Two studies focused on ascending aorta segmentation on CTPA (6) and non-contrast CT (20). Employing a 2D Gaussian smoothing filter to reduce noise prior to segmentation on resampled higher-resolution images can preserve the images’ resolution and quality and achieved higher DSC score in the ascending aorta segmentation compared to models without Gaussian filters (20).

PA segmentation was performed in two CTPA and two non-CTPA studies. Higher DSC scores were achieved in the CTPA studies (5, 6). Consequently, the CT acquisition might play an important role on the DSC scores. In contrast, only two studies were conducted to segment the PV. Sharobeem achieved a DSC of 0.66 in their 2021 study (8) and Li achieved DSCs of 0.80 and 0.77 by modifying two models in their 2020 study (24). Different reasons can explain the low scores. First, using pre-procedural CT data of patients undergoing transcatheter aortic valve implantation (TAVI), where the parameters employed aimed to enhance the differentiation in the aorta and peripheral vascular anatomical features could negatively affect the anatomical structures located on the right side. This could elucidate the unsatisfactory outcomes observed for PV segmentation. Second, both studies were trained and implemented on elevated heart rate, TAVI, or Total Anomalous Pulmonary Venous Connection (TAPVC) patients. Hence, the CT images were expected to have low resolution. Third, inhomogeneous contrast enhancement and beam hardening artefacts were reported to decrease the images’ resolution and affect the segmentation quality (8).

Different limitations have been mentioned in the previous studies including that DL models can mitigate small mistakes in segmentation but cannot avoid observers’ mistakes in the model training step (1, 2). Most of the studies developed their models and tested them in single-centre or single-vendor (3, 6, 8, 19). In addition, it was reported that without using contrast media, contouring the borders between the structures can be difficult (11). Furthermore, using only CT axial slices to train the model can produce lower segmentation quality (5). Image resolution can affect the segmentation quality. Hence, the cardiac phase plays an important role. For example, LV and LVM segmentation can be difficult in the end-systolic phase (7, 21). Some limitations were reported in dealing with patients’ data. For instance, acquiring CT data pre-procedural in TAVI patients can affect the images’ resolution of right-side cardiac anatomical structures (8). Another example in aortic dissection patients where a large tear in the aorta can be difficult to be segmented with DL models (14). Elevated heart rate or atrial fibrillation patients were reported to negatively affect the images’ resolution, producing lower DSC scores (10). DL models using hinge points to find the structures’ borders may negatively affect the estimation, resulting in overestimation in segmentation (19).

Based on the previous limitations, the included studies’ authors recommended future work that can overcome the mentioned drawbacks and increase the quality of DL models in segmentation. Multi-centres involving a high number of patients are recommended to include more anatomical structures’ features and more ethnicities (3, 6, 8, 19). Using a classifier combined with the DL model can reduce the overestimation in segmentation (11). Training the model with different CT acquisitions for instance CT images with and without contrast media can enhance the DSC scores (11). Moreover, training the model with three CT planes (axial, sagittal, and coronal) might improve the segmentation's quality (5).

Based on the previous studies’ limitations and solutions, we reported that there was no study that developed a supervised DL model that can be used on different CT acquisitions and segment different anatomical structures at the same time. In addition, in Figure 5B, only 19% of the studies validated or tested their results on external data and most of the studies used single-centre dataset to develop their models. Furthermore, 56% of the studies have not reported the definition of ground truth reference standard and 37.5% have not stated the used annotation tools. The study design was clearly reported in only 69% of the studies. Hence, we recommend developing a supervised DL model that overcomes the previous limitations by training, testing, and validating the model on different cohorts or among multi-centre, multi-ethnicities, and multi-vendors. By implementing this, the lack of data, external validation and clinical testing can be avoided, resulting in a more accurate model that can be applicable to different cohorts worldwide. It is highly recommended to follow the CLAIM guidelines to cover most of the details to encourage other researchers to build new models in the field and avoid any previous mistakes. In addition, we recommend making DL models and codes available for other researchers to test, adapt and improve. Furthermore, developing an accurate and fast model that can be applied on different CT acquisitions and used to segment different cardiac structures at the same time can make an improvement on DL CT applications, which can be reflected in patients’ diagnosis and treatment in clinical practice.

## Study limitations

Our work had several limitations. First, this systematic review focused solely on AI segmentation tools on CT imaging, where the inclusion criteria were rather narrow (DL tools with supervised training only). Second, the formal meta-analysis of the DSC results was not performed due to heterogeneity between the studies and applied models. Third, despite our efforts to comprehensively identify published DL cardiac CT segmentation studies, it's important to acknowledge that there is an amount of relevant research in the form of unpublished work, preprints, and materials presented at technical conferences that we may have overlooked. Finally, even with the use of structured quality assessment tools (i.e., CLAIM), there is still a degree of subjectivity involved in assessing the quality of reports.

## Conclusion

This systematic review identified few studies presenting supervised DL tools for the segmentation of cardiac structures on CT. The studies demonstrated considerable variability regarding the types of structures being segmented. The different models yielded good DSC scores for most of the major cardiac structures, however, none could be applied on different CT acquisitions and segment different anatomical structures at the same time. The studies were limited by the nature of their training data, inconsistent ground truth definitions and lack of testing on external data. This systematic review highlights the potential for DL tools in evaluation of the heart on CT and identifies shortcomings in study design and reporting that should be addressed to aid advancement of the field.

## Data Availability

The original contributions presented in the study are included in the article/**Supplementary Material**, further inquiries can be directed to the corresponding authors.

## Supplementary material

Supplementary Table S1

Embase and Medline used search terms.

Supplementary Table S2

Full studies’ compliance with CLAIM checklist.

Supplementary Table S3

DSC scores for each segmented cardiac structure.

Supplementary Figure 1

Bar charts showing the included studies were classified into different segmented cardiac structures. (**F**) and (**H**) showing the results of Sharkey et al. (6), the only study focused on RVM and descending aorta, respectively.
