# Supplementary material for: Advancements in cardiac structures segmentation: a comprehensive systematic review of deep learning in CT imaging
Source: Front Cardiovasc Med. 2024 Jan 22;11:1323461. doi: 10.3389/fcvm.2024.1323461 (PMC10839106; doi:10.3389/fcvm.2024.1323461)

Supplementary materials

Supplemental Table 1. Embase and Medline used search terms.

1	((convolutional or neural) adj1 network).ab,ti.
2	((deep or supervised or unsupervised or machine) adj1 learning).ab,ti.
3	(AI or (Artificial adj1 Intelligence) or Algorithm).ab,ti.
4	exp machine learning/
5	(CT or CTPA).ab,ti.
6	(Computer assisted tomography or Computer Tomogrpahy ME).ab,ti.
7	1 or 2 or 3 or 4
8	(heart or cardi* or coronar*).ab,ti.
9	exp computer assisted tomography/
10	5 or 6 or 9
11	7 and 8 and 10
12	Limit 11 to English language
13	Limit 12 to human
14	Limit 13 to yr=”2013 -Current”
15	Remove duplicates from 14

Supplemental Table 2. Full studies’ compliance with CLAIM checklist.

	No.	Criteria	Domain	Yes (%)	No (%)
**Title & abstract**					
Title	1	Identification as a study of AI methodology, specifying the category of technology used (e.g., deep learning)	Study description	100	0
Abstract	2	Structured summary of study design, methods, results, and conclusions	Study description	100	0
**Introduction**					
Introduction	3	Scientific and clinical background, including the intended use and clinical role of the AI approach	Study description	100	0
	4	Study objectives and hypotheses	Study description	100	0
**Methods**					
Study design	5	Prospective or retrospective study	Study description	68.75	31.25

	6	Study goal, such as model creation, exploratory study, feasibility study, non-inferiority trial	Study description	100	0
Data sources	7	Data sources	Dataset description	100	0
	8	Eligibility criteria: how, where, and when potentially eligible participants or studies were identified (e.g., symptoms, results from previous tests, inclusion in registry, patient-care setting, location, dates)	Dataset description	100	0
	9	Data pre-processing steps	Dataset description	87.5	12.5
	10	Selection of data subsets, if applicable	Dataset description	56.25	43.75
	11	Definitions of data elements, with references to Common Data Elements	Dataset description	100	0
	12	De-identification methods	Dataset description	31.25	68.75
	13	How missing data were handled	Dataset description	25	75
Ground truth reference standard	14	Definition of ground truth reference standard, in sufficient detail to allow replication	Dataset description	43.75	56.25
	15	Rationale for choosing the reference standard (if alternatives exist)	Dataset description	6.25	93.75
	16	Source of ground-truth annotations; qualifications and preparation of annotators	Dataset description	100	0
	17	Annotation tools	Dataset description	62.5	37.5

	18	Measurement of inter- and intrarater variability; methods to mitigate variability and/or resolve discrepancies	Dataset description	25	75
Data Partitions	19	Intended sample size and how it was determined	Dataset description	0	100
	20	How data were assigned to partitions; specify proportions	Dataset description	87.5	12.5
	21	Level at which partitions are disjoint (e.g., image, study, patient, institution)	Dataset description	37.5	62.5
Model	22	Detailed description of model, including inputs, outputs, all intermediate layers and connections	Model description	100	0
	23	Software libraries, frameworks, and packages	Model description	81.25	18.75
	24	Initialization of model parameters (e.g., randomization, transfer learning)	Model description	93.75	6.25
Training	25	Details of training approach, including data augmentation, hyperparameters, number of models trained	Model description	100	0
	26	Method of selecting the final model	Model description	50	50
	27	Ensembling techniques, if applicable	Model description	N/A	N/A
Evaluation	28	Metrics of model performance	Model performance	100	0
	29	Statistical measures of significance and uncertainty (e.g., confidence intervals)	Model performance	81.25	18.75
	30	Robustness or sensitivity analysis	Model performance	25	75

	31	Methods for explainability or interpretability (e.g., saliency maps), and how they were validated	Model performance	93.75	6.25
	32	Validation or testing on external data	Model performance	18.75	81.25
**Results**					
Data	33	Flow of participants or cases, using a diagram to indicate inclusion and exclusion	Dataset description	68.75	31.25
	34	Demographic and clinical characteristics of cases in each partition	Dataset description	56.25	43.75
Model performance	35	Performance metrics for optimal model(s) on all data partitions	Model performance	100	0
	36	Estimates of diagnostic accuracy and their precision (such as 95% confidence intervals)	Model performance	N/A	N/A
	37	Failure analysis of incorrectly classified cases	Model performance	N/A	N/A
**Discussion**					
Discussion	38	Study limitations, including potential bias, statistical uncertainty, and generalizability	Model performance	93.75	6.25
	39	Implications for practice, including the intended use and/or clinical role	Model performance	81.25	18.75
**Other information**					
Other information	40	Registration number and name of registry	Study description	0	100
	41	Where the full study protocol can be accessed	Study description	0	100
	42	Sources of funding and other support; role of funders	Study description	81.25	18.75

N/A: not applicable.

Supplemental Table 3: DSC scores for each segmented cardiac structure.

Segmented structure	Study	CT acquisition	DSC scores
LA	Bruns 2020	CT - & CCT	0.92
	Chen 2020	PVCT	0.96 *
	Aquino 2022	CTA	0.94
	Sharobeem 2021	CCT	0.94
	Abdulkareem 2022	CCT	0.89
	Van den Oever 2022	LDCT	0.82
	Li 2020	LDCT	GAB= 0.83<br>SCAB= 0.81
	Sharkey 2022	CTPA	DL1 – Internal cohort= 0.90<br>DL2 – Internal cohort= 0.91<br>DL1 – External cohort= 0.88<br>DL2 – External cohort= 0.87
	Kazi 2023	CTA	Unified-image-volume= 0.86<br>Regional patch-volumes= 0.87
	Yao 2023	CTA	0.79
LV	Gupta 2021	CTA	0.97
	Bruns 2020	CT - & CCT	0.90
	Aquino 2022	CTA	0.94
	Sharobeem 2021	CCT	0.85
	Van den Oever 2022	LDCT	0.92
	Guo 2021	CCT	0.96
	Sharkey 2022	CTPA	DL1 – Internal cohort= 0.91<br>DL2 – Internal cohort= 0.91<br>DL1 – External cohort= 0.90<br>DL2 – External cohort= 0.89
	Yao 2023	CTA	0.82
LVM	Guo 2020	CCTA	0.92
	Bruns 2020	CT - & CCT	0.84
	Aquino 2022	CTA	0.93
	Sharobeem 2021	CCT	0.93
	Sharkey 2022	CTPA	DL1 – Internal cohort= 0.82<br>DL2 – Internal cohort= 0.83<br>DL1 – External cohort= 0.82<br>DL2 – External cohort= 0.83
RA	Bruns 2020	CT - & CCT	0.91

	Aquino 2022	CTA	0.92
	Sharobeem 2021	CCT	0.88
	Van den Oever 2022	LDCT	0.80
	Sharkey 2022	CTPA	DL1 – Internal cohort= 0.89<br>DL2 – Internal cohort= 0.90<br>DL1 – External cohort= 0.84<br>DL2 – External cohort= 0.88
	Yao 2023	CTA	0.83
**RV**	Bruns 2020	CT - & CCT	0.92
	Aquino 2022	CTA	0.93
	Sharobeem 2021	CCT	0.82
	Van den Oever 2022	LDCT	0.88
	Sharkey 2022	CTPA	DL1 – Internal cohort= 0.92<br>DL2 – Internal cohort= 0.92<br>DL1 – External cohort= 0.90<br>DL2 – External cohort= 0.91
	Yao 2023	CTA	0.78
**RVM**	Sharkey 2022	CTPA	DL1 – Internal cohort= 0.58<br>DL2 – Internal cohort= 0.58<br>DL1 – External cohort= 0.58<br>DL2 – External cohort= 0.59
**Ascending aorta**	Bruns 2020	CT - & CCT	0.94
	Sharkey 2022	CTPA	DL1 – Internal cohort= 0.92<br>DL2 – Internal cohort= 0.92<br>DL1 – External cohort= 0.93<br>DL2 – External cohort= 0.91
**Descending aorta**	Sharkey 2022	CTPA	DL1 – Internal cohort= 0.91<br>DL2 – Internal cohort= 0.91<br>DL1 – External cohort= 0.87<br>DL2 – External cohort= 0.87
**PA**	Bruns 2020	CT - & CCT	0.86
	Sharobeem 2021	CCT	0.88
	Yuan 2023	CTPA	0.94
	Sharkey 2022	CTPA	DL1 – Internal cohort= 0.93<br>DL2 – Internal cohort= 0.93<br>DL1 – External cohort= 0.91<br>DL2 – External cohort= 0.93
	Yao 2023	CTA	0.67
**PV**	Sharobeem 2021	CCT	0.66
	Li 2020	LDCT	GAB= 0.80<br>SCAB= 0.77

**SVC**	Sharobeem 2021	CCT	0.63
**Aorta**	Chen 2022	CCT & CTA	CCT= 0.95<br>CTA= 0.95
	Sharobeem 2021	CCT	0.92
	Yao 2023	CTA	0.82
**Dissected aorta**	Lyu 2021	CTA	0.92
**Whole heart**	Van den Oever 2022	LDCT	0.96
**Heart chambers & LVM**	Bruns 2022	CCT	3D in 12 patients= 0.89<br>2D in 81 patients= 0.89
**Coronary sinus**	Sharobeem 2021	CCT	0.60
**Left coronary cusp (LCC)**	Astudillo 2020	CCT	Not reported
**Non-coronary cusp (NCC)**	Astudillo 2020	CCT	Not reported
**Right coronary cusp (RCC)**	Astudillo 2020	CCT	Not reported
**Left-coronary ostium (LCO)**	Astudillo 2020	CCT	Not reported
**Right-coronary ostium (RCO)**	Astudillo 2020	CCT	Not reported
**Myocardium**	Yao 2023	CTA	0.77

*Intersection Over Union (IuO) was converted to Dice Similarity Coefficient (DSC) based on previously published formulas (4).

Cardiac CT (CCT), CT Angiography (CTA), Coronary CT Angiography (CCTA), Pulmonary Vein CT (PVCT), Low Dose CT (LDCT).

Supplemental Figure 1. Bar charts showing the included studies were classified into different segmented cardiac structures. **(F)** and **(H)** showing the results of Sharkey 2022 (6), the only study focused on RVM and descending aorta, respectively.

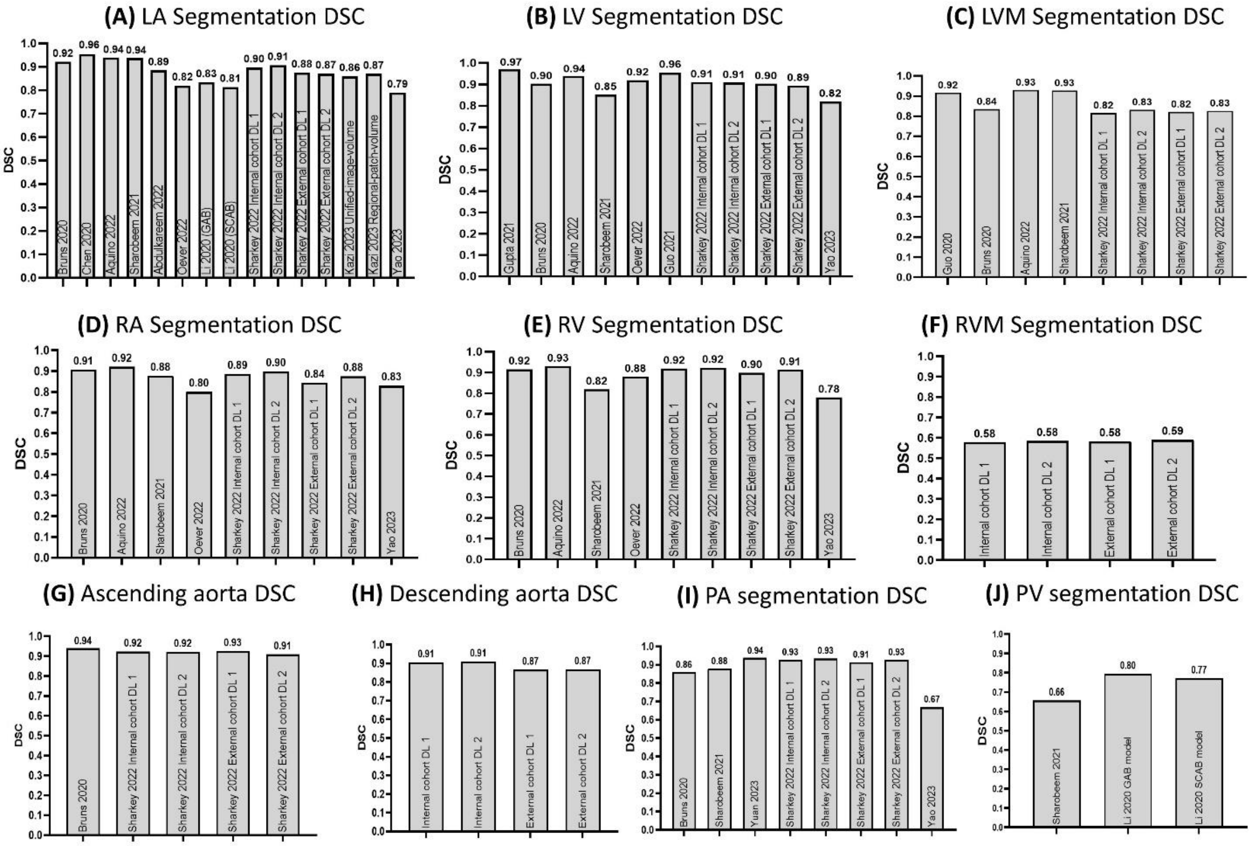

Supplement: Supplementary file 1 [file Datasheet1.pdf]
